# Lipid–Protein Interplay in the Regulation of Receptor Tyrosine Kinases

**DOI:** 10.3390/cells14231836

**Published:** 2025-11-21

**Authors:** Mattia Domenichini, Anna Gogna, Camilla Maggi, Elisa Moreschi, Anna Ventura, Martina Codibue, Elisabetta Grillo, Michela Corsini, Stefania Mitola

**Affiliations:** 1 Department of Molecular and Translational Medicine, University of Brescia, 25123 Brescia, Italy; m.domenichini001@unibs.it (M.D.); anna.gogna@unibs.it (A.G.); c.maggi002@studenti.unibs.it (C.M.); e.moreschi005@studenti.unibs.it (E.M.); anna.ventura@unibs.it (A.V.); martina.codibue@unibs.it (M.C.); elisabetta.grillo@unibs.it (E.G.); 2 Mechanobiology Center, University of Brescia, 25123 Brescia, Italy

**Keywords:** RTK, lipids, membrane microdomains, lipid-targeting therapies

## Abstract

**Highlights:**

**What are the main findings?**

1. Specific lipid–protein interactions tightly regulate RTKs’ activity and function in physiological and pathological contexts.
2. Plasma membrane lipid heterogeneity spatially and mechanically controls RTK trafficking, clustering, and downstream signaling.

**What is the implication of the main finding?**

1. A deeper understanding of lipid–RTK crosstalk is required to resolve the spatiotemporal complexity of this regulatory mechanism.
2. Lipid-dependent modulation of RTKs offers promising therapeutic approaches.

**Abstract:**

Receptor tyrosine kinases (RTKs), a class of membrane proteins involved in several physiological processes such as growth, survival, angiogenesis, and differentiation, are profoundly influenced by the microenvironment, particularly by surrounding lipids. Lipids coordinate RTK life cycle at multiple steps. First, receptor lipidation is a key post-translational modification for receptor-targeting localization. Then, RTK dimerization and activation are regulated by membrane-enriched lipids like phosphatidylserine and phosphoinositides, gangliosides, and Cholesterol, which directly engage RTK juxtamembrane domain or cytoplasmic tail. Eventually, lipids spatially organize RTK signaling within Cholesterol- and sphingolipid-enriched membrane microdomains. These membrane rafts act as dynamic “signalosomes” coordinating receptor clustering, endocytosis, and recycling. Perturbations in lipid composition remodel raft architecture and alter RTK behavior, contributing to pathological conditions such as cancer, metabolic, and neurodegenerative disorders. Emerging lipid-targeted therapies offer a promising way to enhance RTK-directed therapies. This review aims to explore how specific lipid species and membrane domains modulate RTK activation, clustering, and endocytic recycling. By bridging biochemical and pathological perspectives, we discuss how membrane lipid composition reshapes RTK signaling in physiology and pathology, pointing to emerging opportunities for lipid-focused therapeutic modulation.

## 1. Introduction

The plasma membrane is a dynamic structure essential for maintaining cellular homeostasis. Beyond providing a physical barrier between the intracellular and extracellular compartments, it functions as an active platform that orchestrates nutrient transport, cell communication, and signal transduction. These processes are largely governed by the molecular organization of the membrane, which is composed of lipids, proteins, and carbohydrates in variable proportions. Among them, lipids are not only structural elements but also regulators of membrane architecture and signaling, as they form specialized microdomains and directly modulate protein functions including tyrosine kinase receptors (RTKs). RTKs, by binding extracellular molecules, including growth factors, hormones, and cytokines, mediate key physiological processes including the metabolic regulations, proliferation, differentiation, migration, and angiogenesis. Their deregulation contributes to the onset and progression of numerous human diseases, including metabolic disorders and cancer [1]. Traditionally, RTK activity has been studied in terms of ligand binding, dimerization, and intracellular signaling cascade. However, increasing evidence shows that their structure, localization, clustering, and activation are profoundly influenced by the lipid environment of the plasma membrane.

Here, we provide an overview of membrane lipid composition and organization and discuss how specific lipid species and membrane domains regulate RTK activation, signaling, and trafficking. Eventually, this review aims to explore novel lipid-targeted therapies and their efficacy on dysregulated RTK activities. By highlighting the interplay between lipids and RTKs, we illustrate how membrane composition contributes to the fine-tuning of receptor function in physiology and disease.

## 2. Cell Membrane

Eukaryotic cell membrane, also known as plasma membrane, is a highly complex, dynamic, and multifunctional structure that plays a key role in the architecture and physiology of cells. Plasma membrane ensures compartmentalization, separating the internal and external environment of the cell, and mediates cell-to-cell communication and intracellular signals [2]. The ability to perform multiple functions is closely related to the heterogeneity of its composition. Structurally, it comprises approximately 50% of proteins, 40% of lipids, and 5–10% of carbohydrates. Lipids form a bilayer that acts as a stable selective transport barrier. Proteins confer functional properties such as signal transduction, transport, and cell adhesion, while carbohydrates, covalently associated to lipids or proteins, facilitate molecular and cellular interactions [3].

### 2.1. Membrane Lipid Composition

Lipids constitute the bulk components of the cell membrane. They are hydrophobic or amphipathic molecules and comprise, among others, fatty acids (FAs), sterols, mono-, di-, triacylglycerols (M-D-TAGs), as well as phospholipids (PLs) [4]. Glycerophospholipids (GPLs), sphingolipids (SLs), and sterols are the principal components of membranes. GPLs represent the predominant structural lipids, consisting of two hydrophobic FAs esterified to a glycerol backbone which is further associated with a phosphate-containing head group. The phosphate group can be modified by various polar groups such as choline, ethanolamine, serine, or inositol, defining the specific class of GPL. GPL structural characteristics, including the length and saturation of fatty acid chain, together with the nature of the phosphate-head group, critically influence membrane functional properties [2]. SLs are characterized by a hydrophobic backbone derived from sphingosine which is N-acylated with a fatty acid to form ceramide. This ceramide core can be further modified through the addition of different polar head groups. The predominant SL in mammalian membranes is the sphingomyelin (SM), characterized by a phosphorylcholine head group. SM accounts for approximately 2–15% of the total PL content, depending on the cell type. Another important SL subclass includes glycosphingolipids (GSLs), which are based on glucosylceramide or galactosylceramide and are characterized by their conjugated mono-, di-, or oligosaccharide chains. Among them, gangliosides (GMs), composed of a ceramide backbone linked to a complex sugar chain containing one or more sialic acids, are relevant constituents of the membrane, and involved in its structural organization and lateral compartmentalization [2]. Sterols, or steroid alcohols, are also present in plasma membranes. Cholesterol (Chol) is the principal sterol species comprising up to 50% of total membrane lipids. Chol consists of a rigid tetracyclic steroid nucleus, a hydroxyl group on the A-ring, and a short-branched hydrophobic tail on the D-ring. The amphipathic nature allows Chol to have extensive interactions with both PLs and SLs and makes it a central component of lipid rafts. The heterogeneity of the membrane lipid composition contributes to defining its structural organization and consequently impacts its functional and mechanical properties, including permeability, fluidity, and curvature. Indeed, when in a monolayer, lipids, depending on their type, tend to adopt the curvature that minimizes the free energy of their system (spontaneous curvature); since in the plasmatic membrane they are forced into a different position, the membrane accumulates a mechanical stress energy (curvature elastic stress) that results in the resistance to bending deformations [5,6].

Membrane lipid composition is dynamically regulated through both endogenous lipid biosynthesis and dietary intake. Cells synthesize FAs, GPLs, SLs, and sterols via highly coordinated metabolic pathways, which are tightly controlled by metabolites’ availability and signaling cues. At the same time, dietary lipids provide an important external source of structural building blocks. For instance, the proportion of saturated versus unsaturated FAs in the diet directly affects the acyl chain composition of membrane PLs, hence the unsaturated FAs increase membrane fluidity, while saturated ones pack closely, raising membrane rigidity [7]. Essential FAs like linoleic and α-linolenic acid are incorporated into the membranes and further elongated or desaturated to form polyunsaturated fatty acids (PUFAs). Thus, the interplay between endogenous lipid synthesis and exogenous dietary lipid supply provides a means for cells to adapt membrane properties to environmental conditions, ensuring proper function of membrane-associated processes such as vesicle trafficking, receptor activation, and signal transduction [8].

### 2.2. Membrane Lipid Structure and Organization

The plasma membrane exhibits a non-homogeneous lipid composition, forming a dynamic and heterogeneous bilayer. According to the fluid mosaic model proposed by Singer and Nicolson in 1972, lipids and proteins possess lateral mobility, conferring flexibility and dynamic properties to the membrane. In addition to its composition and structural plasticity, the plasma membrane constantly adapts to multidirectional mechanical stresses [4]. Therefore, lipids actively regulate membrane architecture, remodeling, intracellular trafficking, and signaling in mammalian cells. Specific lipids, such as phosphoinositides, SLs, and Chol, act as molecular signals and structural scaffolds, recruiting and activating proteins involved in membrane budding, vesicle fusion, and trafficking. Protein domains including Pleckstrin Homology (PH), Phox homology (PX) and FYVE (Fab-1, YGL023, Vps27, and EEA1), and Second Cysteine-rich domains recognize specific lipid head groups, targeting signaling molecules to define membrane compartments and coordinating trafficking events. Lipid transfer proteins, including oxysterol-binding protein (OSBP) and OSBP-related proteins (ORPs), further contribute to membrane dynamics by mediating non-vesicular lipids exchange at membrane contact sites, linking lipid metabolism to intracellular signaling pathways [9].

Within certain regions of the plasma membrane, lipid–lipid interaction can lead to the formation of specific compartments, referred to as membrane microdomains, membrane rafts, or lipid rafts. According to the ‘raft hypothesis’, they are highly dynamic nanoscale liquid-ordered domains in the plasma membrane, which are enriched in Chol and SLs and serve for vesicle docking and signal transduction [10]. These microdomains, small (10–100 nm), dynamic, and tightly packed are embedded within a disordered PLs bilayer [11]. Despite heterogeneity in composition, lipid rafts consistently contain high levels of SM and GSLs. The unique GM structure enables the formation of nanoscopic assemblies, called “ganglioside nanodomains”, which contribute to raft integrity and function. Within lipid rafts, Chol inserts between SL saturated hydrocarbon chains, stabilizing their compact structures. The ordered structure of rafts is further sustained by the high saturation of lipid acyl chains, in contrast with the unsaturated PLs of non-raft domains. Due to this specific composition, rafts are insoluble in cold non-ionic detergents (e.g., 1% Triton X-100), earning them the name “detergent-resistant membranes”. Functionally, lipid rafts act as signal hubs. By selectively recruiting or excluding specific kinases, phosphatases, and other signaling molecules, they spatially and temporally regulate signaling dynamics in response to internal and external stimuli. Raft clustering enhances signal specificity and strength, preventing premature protein degradation and regulating oligomerization and activation of membrane proteins [11].

Lipid rafts are broadly classified into planar rafts and caveolae. Planar rafts are ubiquitous across cell types and are stabilized by integral proteins such as flotillin1 and 2. Caveolae, on the other hand, are flask-shaped membrane invaginations enriched in specific cell types including muscle cells, adipocytes, endothelial cells, and fibroblasts. They are formed by caveolins, a hairpin-loop scaffolding protein localized in the inner leaflet of the membrane [12]. Caveolae mediate caveolin-dependent endocytosis and the internalization of GSLs, GPI-anchored proteins, extracellular ligands, bacterial toxins, and viruses [13]. Despite extensive studies, a common consensus on the “raft hypothesis” is still lacking. Much of the current understanding derives from model membranes and isolated systems, which fail to capture the dynamic organization and functional relevance of rafts in their native cellular environment. Direct visualization of these domains in living cells remains technically challenging due to their small size, transient nature, and sensitivity to membrane composition. Further methodological advances are therefore essential to elucidate the role of lipid rafts and caveolae under physiological and pathological conditions [10].

### 2.3. Membrane Lipid Functions

Membrane lipids fulfill three major functions: energy storage, compartmentalization, and signaling. First, due to their relatively reduced state, lipids are well-suited for long-term energy storage. The amphipathic nature of lipids underlies their ability to spontaneously assemble into bilayers in aqueous environments. The hydrophobic effect drives the self-association of acyl chains, while hydrophilic head groups interact with the aqueous milieu, resulting in bilayer formation. This structural organization enables cellular compartmentalization which, in turn, allows the segregation of biochemical reactions, the enhancement of metabolic efficiency and the restriction of non-specific diffusion of metabolites. In addition to acting as a selective barrier, membranes support dynamic processes, including budding, tubulation, fission, and fusion, which are directly governed by lipid physical properties. These processes are essential for cell division, intracellular trafficking, and organelle biogenesis [14]. Moreover, the lateral organization of membrane lipids promotes the spatial segregation of protein complexes, enabling the formation of functional microdomains. Lipids also serve as intracellular transducers in several pathways, coordinating the assembly and activity of effector complexes [15]. Lipid-mediated signaling occurs at multiple membrane levels, both extracellular and intracellular, reflecting its spatial and functional complexity. Extracellularly, bioactive lipids including sphingosine-1-phosphate (S1P), prostaglandins, and lysophosphatidic acid (LPA) secreted in association with extracellular vesicles or bound to carrier proteins interact with lipid-sensitive G protein-coupled receptors (GPCRs) or RTKs, modulating receptor signaling [16]. These observations highlight the critical role of membrane lipids not only as structural entities but also as dynamic regulators of signal transduction. In the following paragraphs we summarize the dynamic of RTK–lipid interactions to unravel the spatial logic of membrane-associated signaling networks.

## 3. Receptor Tyrosine Kinases

Both in eukaryotes and in prokaryotes, protein phosphorylation is a key factor for signal transduction. While in prokaryotes the phosphorylation signaling is mainly mediated by phospho-histidine, in eukaryotes, the phosphorylation of serine, threonine, and tyrosine is the main transduction pathway. The whole process is regulated by the balance of tyrosine kinases and tyrosine phosphatases. RTKs are codified by 58 genes and comprise 20 subfamilies of receptors. Despite considerable diversity among these receptors, they all share a highly conserved tyrosine kinase domain (KD), which provides the core phosphotransferase activity essential for their function [17]. Tyrosine phosphorylation consists of the addition of a phosphate group to a tyrosine residue within the sequence of a target protein, triggering its structural and functional modifications. Tyrosine phosphatases balance this post-translational modification by hydrolyzing the phosphate group. All KDs have evolved from a common precursor, which defined the boundaries of what is now recognized as one of the largest gene families encoded by eukaryotic genomes. RTKs are transmembrane molecules involved in several physiological processes like survival, migration and proliferation, angiogenesis, embryo development, and cellular metabolism [1]. Besides their structural similarities, RTKs also converge on common downstream signaling pathways. Upon ligand binding, they undergo dimerization, triggering trans-autophosphorylation on different tyrosine residues. The resulting pTyr thus engages adaptor proteins and enzymes, containing SH2 and PTB domains. These adaptor molecules, in turn, transduce the signal to downstream signaling effectors, creating a cascade that eventually results in a cellular response. Particularly, most RTKs drive their biological activity through two main pathways: the RAS/MAPK and the PI3K/AKT pathways, both regulating cell proliferation, migration, and survival [18]. Another key pathway that can be activated is the one headed by Phospholipase C gamma (PLCγ), which modulates calcium signaling and cytoskeletal reorganization [19].

### RTKs Structure and Activation

RTKs are structured in extracellular, transmembrane, and cytoplasmic domains which contain the catalytic domain. Depending on the RTK class, the extracellular region is provided with a binding domain that consists of a variable number of repeated sequences including immunoglobulin-like domains enriched in Leu and Cys, fibronectin type III domains, and/or others. These are characterized by highly diverse ectodomains for ligand recognition and the following receptor dimerization [1]. The transmembrane domain (TMD) is composed of a hydrophobic α-helix chain of about 20 amino acids responsible for the stability of the full-length dimer and the maintenance of a signaling-competent structure [20]. Beyond a mere structural anchor, the TMD plays an active role in receptor activation by transmitting ligand-induced conformational changes from the extracellular to the intracellular domains. This transmission often involves rotational or lateral movements of the α-helices within the membrane bilayer, aligning the cytoplasmic KDs for effective dimerization and autophosphorylation [20]. The TMD is linked with the catalytic domain through a small sequence, called juxtamembrane domain (JMD). The JMD comprises 40–80 amino acids including phosphorylable sites and docking motifs like the Fibroblast growth factor receptor substrate 2α (FRS2α). The JMD regulates the RTK autoinhibition by stabilizing the KD in an inactive conformation, interfering with the activation loop or αC-helix, and occluding substrate or ATP access. The JMD region interacts with membrane lipids, affecting KD orientation and clustering. Moreover, a glycan binding sequence within the JMD of RTKs regulates cell surface localization and intracellular STAT signaling [21]. Finally, the KD region, consisting of 250–300 amino acids, transfers the γ-phosphate of the ATP (or GTP) molecule to various substrates including lipids, sugars, or amino acids. The KD contains a β-stranded N-lobe, connected by a short hinge region to a larger αC-helix and an activation loop (A-loop). ATP binds in the cleft between the N- and C-terminal lobes of the KD [22]. Inside the A-loop, the DFG (Asp, Phe, and Gly) motif interacts with the Mg2+, coordinating the transfer of γ-phosphate of ATP. The DFG motif and the αC-helix are subjected to many conformational changes during enzymatic activation. While the DFG residues are necessary for the kinase activity, the αC-helix is required but not sufficient in the enzymatic activation process. Thus, the two motives are in different conformational states during receptor activation, inhibition, or during the transition phase [23]. The kinase activation process involves several tyrosines spatially and temporally regulated by the 3D folding of the KD. Ordered autophosphorylation of tyrosine in the A-loop of several RTKs has been described, suggesting that kinases regulate their own phosphorylation process by modulating their conformational states [24]. Sequential autophosphorylation of tyrosine in the catalytic core is under kinetic control and it depends on their degree of accessibility resulting from protein folding. In this process, the phosphoryl transfer is the rate-limiting step.

RTKs’ activation can be modulated by the interaction with cellular membrane lipids. 

At the plasma membrane, RTKs such as the Epidermal Growth Factor Receptor (EGFR) and the Vascular Endothelial Growth Factor Receptor (VEGFR) dynamically localized within lipid raft microdomains [25,26], modulating receptor signaling. RTK activation induces lipid-derived second messengers such as diacylglycerol and inositol trisphosphate (IP_3_), which propagate the signal by activating downstream effectors [27]. Lipid metabolites translocate to the nucleus, influencing gene expression and chromatin remodeling through kinase cascade or direct action [28]. Upon ligand interaction and internalization, RTKs continue to signal from endosomal compartments, where lipid composition regulates the fidelity and duration of the downstream signaling. 

In the next sections, we thoroughly described how lipids can modulate RTKs’ life cycles at different stages, influencing the receptor’s activation. 

## 4. Role of Lipids in the Maturation, Activation, and Recycling of RTKs

RTK life cycle is a multistep process which relies on stringent regulatory mechanisms. Lipids are key regulators of each step, from protein maturation to receptor signaling and recycling, securing RTKs’ correct localization, interactions, and signaling. First, receptor maturation is a necessary step for the correct protein functionality. Within the endoplasmic reticulum and the Golgi apparatus, RTKs acquire their mature conformation and undergo post-translational modifications, including glycosylation, protein–protein interactions, and lipidation, which collectively ensure proper receptor folding, stability, and trafficking to functional membrane compartments. RTK activation typically requires ligand binding, which induces receptor homo- or heterodimerization. A critical regulatory step for RTK activity is the control of receptor abundance and distribution at the plasma membrane, which influences receptor clustering, dimerization, and interaction with molecular partners. Membrane composition and organization are therefore critical in shaping RTK dimerization and signaling. The plasma membrane acts as a dynamic molecular platform that hosts and coordinates essential cellular processes. It is structured into distinct functional domains, often supported by cortical actin and adaptor proteins [29]. In this context, RTK activation and clustering are strongly influenced by interactions among cytoskeletal elements, transmembrane receptors, and lipids within specialized microdomains [30].

Precise spatial and temporal control of RTKs is essential for physiological cellular functions, whereas its disruption results in aberrant activation. These mechanisms include gain- or loss-of-function mutations [31], genomic amplification, and chromosomal rearrangements. Notably, alterations in membrane lipid composition also modulate RTK signaling in several pathological conditions such as congenital disorders, diabetes, and cancer [32].

### 4.1. RTK Lipidation

**Figure 1:**
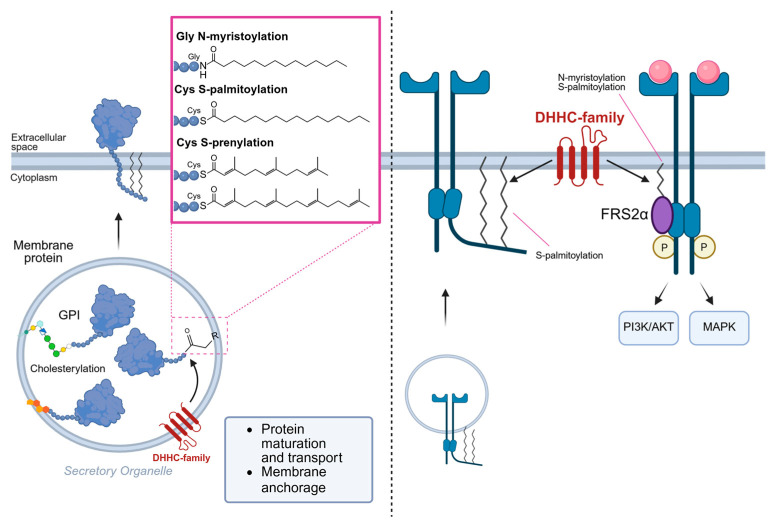
Process of protein lipidation and their effects on RTK behavior.

Among others, lipidation is one of the post-translational modifications occurred in RTKs. Lipidation increases protein complexity by regulating trafficking, membrane association, stability, conformation, and interaction networks in response to extracellular and intracellular stimuli [33]. To date, several lipids have been identified associated with proteins, including FAs, lipoic acids, isoprenoids, sterols, PLs, GPI anchors, and lipid-derived electrophiles (LDEs) [34]. Protein lipidation events are generally classified according to the subcellular compartment in which they occur. Modification on the cytoplasmic side include C16 palmitate covalently linked to Cys (S-palmitoylation) or Ser (O-palmitoylation), C14 myristic acid bound to glycine residues (N-myristoylation) and C15 farnesyl or C20 geranylgeranyl isoprenoid lipid linked to Cys (S-prenylation), while lumenal modifications, such as GPI anchoring and cholesterylation, take place within secretory organelles [33]. Direct and indirect lipidation of RTKs have been described. In particular, EGFR palmitoylation at Cys1025/1034 tethers its intracellular domain to the membrane, limiting ligand-induced autophosphorylation [35]. Conversely, palmitoylation at Cys1122 increases EGFR activation, promoting receptor turnover [36]. c-MET palmitoylation is essential for Golgi–PM trafficking and stability [37]. The DHHC enzyme family catalyzes palmitoylation, and reduced EGFR palmitoylation through DHHC20 depletion suppressing tumorigenesis in mouse lung cancer models, suggesting aberrant DHHC activity in cancer [36]. Beyond these specific examples involving RTKs, the behavior of many other membrane receptors is also modulated by lipidation. S-palmitoylation guides partitioning into lipid rafts, and controls internalization, recycling, and coupling efficiency, as demonstrated in GPCRs such as the β_2_-adrenergic receptor and in T cell receptor signaling complexes [38]. Other proteins are lipidated during their maturation. For example, C-prenylation of small GTPases such as Ras and Rab increases the proteins’ hydrophobicity, allowing the membrane anchorage and ensuring spatial organization of receptor-proximal cascades; inhibition of prenylation disrupts these pathways, leading to protein mis-localization [39]. N-myristoylation of kinases and G proteins helps position signaling molecules near the plasma membrane and dynamically regulate receptor engagement. Lipidation also affects adaptor proteins’ signaling, such as FRS2α, whose N-myristoylation and palmitoylation are required for membrane targeting and activation of downstream PI3K/AKT and MAPK pathways also upon RTK activation [40] (Figure 1). In summary, accumulating evidence identifies protein lipidation as a pivotal regulatory layer controlling protein localization, membrane behavior, and signaling efficiency. These findings underscore the need to intensify our efforts to identify potential lipidation sites in RTKs. Indeed, by modulating receptor localization and interaction with downstream effectors, lipidation might likely act as a dynamic switch that coordinates trafficking, activation, and degradation, ultimately shaping the amplitude and duration of RTK-dependent signaling.

### 4.2. Lipid Regulation of RTK Activation and Intracellular Signaling

Lipid–RTK interactions directly regulate receptor dimerization and activation. Comprehensive study of 58 human RTKs showed that JMDs engage anionic lipids such as phosphatidylserine (PS) and phosphatidylinositol-phosphates (PIPs) [41], reorganizing membrane lipid domains and promoting receptor activation [42]. The absence of PIP2 binding to JMD reduces the ability of Epidermal Growth Factor (EGF) to induce EGFR activation [43]. Indeed, Phosphatidylinositol-4,5-bisphosphate (PIP2) supports EGFR and EphA2 homo- and heterodimerization as well as their multimeric assembly. Interestingly, high PIP2 stabilize a non-productive EGFR-EphA2 heteromultimer in which the KD of EGFR sterically blocks EphA2 phosphorylation [44]. Also, GMs affect RTK dimerization and autophosphorylation,: monosialogangliosides generally act as negative regulators, whereas disialogangliosides favor ligand-independent activation [45]. For instance, GM3 inhibits EGFR autophosphorylation by binding its extracellular domain [46]. Altered GM3 levels are linked to cancer progression; their deficiency enhances EGFR activation and epidermoid carcinoma [47]. Moreover, supplementation of exogenous GM3 suppresses tumor angiogenesis by competing with the ligand for VEGFR2 extracellular binding site [48,49]. GM3 also antagonizes the pro-angiogenic effect of ganglioside GD1a, thereby reducing endothelial cell proliferation [50]. In adipocytes, GM3 interacting with Lys in the β-subunit of insulin receptor regulates insulin signaling by altering receptor localization in caveolae [51]. GM1 is a key co-receptor stabilizing the receptor TrkA-NGF complex on cell membrane [51]. GM1 deficiency also contributes to Parkinson’s disease, along with reduced GD1a levels [52]. Extracellular GM1, by binding Fibroblast Growth Factor 2 (FGF2), prevents its interaction with specific receptors, whereas GM1-enriched membranes positively regulate ligand–receptor binding, acting as FGF co-receptor [53].

**Figure 2:**
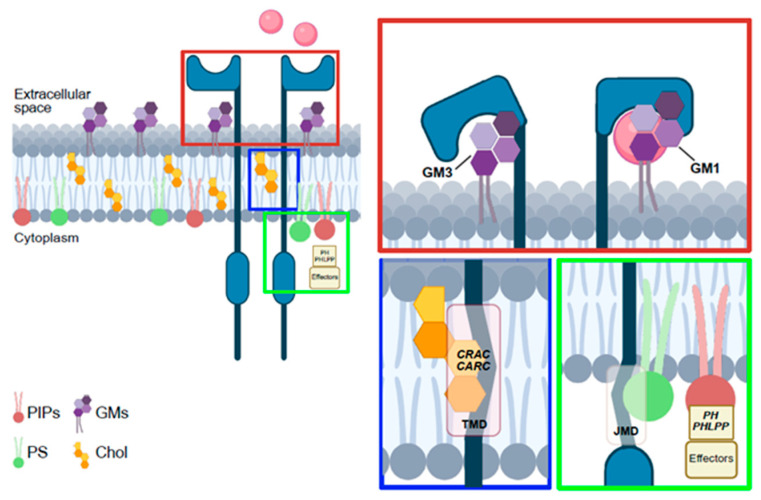
Lipid/RTK interactions in plasma membrane.

Chol interacts directly with specific protein domains, including the Cholesterol Recognition/Interaction Amino Acid Consensus (CRAC) motif and its inverted counterpart (CARC), both localized within the transmembrane domains of various receptors. These motifs share a conserved amino acid pattern, typically consisting of an apolar residue (Leu or Val), followed by one to five residues of any kind, an aromatic residue (Tyr or Phe), another stretch of one to five residues of any kind, and finally a basic residue (Arg or Lys), arranged from the N- to the C-terminus. According to the UniProt database, such sequences are present in several RTKs including Ephrin type-A receptor 3, FGFR1–4, the insulin-like growth factor 1 receptor (IGF1R), ErbB-4, and members of the tropomyosin receptor kinase family [54]. These CARC/CRAC motifs promote the relocation of receptors into Chol-enriched membrane microdomains and are critical for receptor dimerization and phosphorylation. Consistent with this, alterations in CARC/CRAC sequences impair receptors’ dimerization. Chol sensing, and phosphorylation, thereby contribute to various pathological conditions [55]. For instance, the Y433F substitution in Tropomyosin-related kinase B (TRKB) has been associated with several neurodegenerative disorders. Conversely, the functional consequences of the I564V substitution in Ephrin type-A receptor 3 and the F978Y mutation in the insulin receptor remain poorly characterized. Lipids additionally regulate second messengers downstream of RTKs. Growth factors-induced activation of phosphoinositide 3-kinase (PI3K) converts PIP2 to phosphatidyl-inositol-3,4,5-trisphosphate (PIP3), which recruits PH-domain proteins such as serine-threonine kinases Akt and phosphoinositide-dependent kinase 1 (PDK1), enabling Akt phosphorylation by PDK1 [56]. The Akt1 E17K mutation drives growth factor-independent membrane localization and hyperactivation in multiple cancers [57]. Loss of the phosphatase pleckstrin homology domain leucine-rich repeat protein phosphatase (PHLPP) contributes to glioblastoma, metastatic breast cancer, and therapy resistance, as it normally dephosphorylates Akt and represses RTK transcription through chromatin modification [58,59]. Akt activation also requires PS, which enhances PIP3 binding and stabilizes its active conformation. PS depletion impairs Akt signaling and sensitizes cells to apoptosis [60]. Finally, RTK signaling frequently engages the MAPK cascade (Raf, MEK, and ERK), which is also regulated by lipids [61]. Phosphatidic acid and PS facilitate Raf-1 membrane recruitment and activation [62] (Figure 2).

### 4.3. Lipids Spatially Regulate RTKs

The plasma membrane is a heterogeneous structure where proteins and lipids organize into domains with distinct biophysical properties. Here, lipid–protein interactions play a pivotal role in cellular response to environmental challenges and stress conditions. In response to this rapid rearrangement of membrane domains through spatial reorganization and membrane composition, rebalance is a key determinant for cell survival. Moreover, environmental factors such as pH fluctuations, macromolecular crowding, and ion availability add an additional regulatory layer, influencing electrostatic interactions among membrane lipids, the ionization state of lipid headgroups, and membrane mechanics via osmotic stress [63]. Consistent with this, perturbations in lipid–protein coupling have been implicated in several pathologies. In neurodegenerative diseases, aberrant phase behavior of proteins such as tau and TDP-43 leads to abnormal condensate formation and membrane dysfunction. Similarly, metabolic disorders that alter lipid composition can impair condensate dynamics and cellular organization. Membrane lipid composition plays a key role in RTK spatial regulation, and its alteration profoundly impacts intracellular signaling. Many RTKs are enriched in lipid rafts and SL-rich microdomains, which act as signaling platforms by clustering receptors, adaptors, enzymes, and substrates within confined membrane regions. The dynamic remodel of rafts modulates RTK compartmentalization, interaction, and activation [11,64]. The stability of signaling complexes, termed “signalosomes”, within these rafts amplifies downstream pathways that control cell proliferation, differentiation, and apoptosis. The biophysical properties of these microdomains influence receptor clustering, accessibility to co-activating kinases, and protection from phosphatases, thereby shaping the specificity and amplitude of signaling responses. For instance, ligand-induced activation of the TRKB receptor by the brain-derived neurotrophic factor promotes its translocation into lipid rafts through a mechanism requiring the Src-family kinase Fyn; of consequence, disruption of Fyn activity or raft integrity impairs TRKB phosphorylation and PLCγ activation [65]. Similarly, FGFR2 and VEGFR2 localize to lipid rafts [26,64]. Of note, Chol depletion by β-methyl-cyclodextrin (MβCD) selectively abolishes receptor phosphorylation and interaction with their downstream interactors, including FRS2α, PI3K, and Akt [11,66,67]. FGFR2 activation is also spatially regulated by the adaptor growth factor receptor-bound protein 2, which maintains the receptor in a non-raft environment under basal conditions, characterized by lower Chol content, more unsaturated fatty acyl chains, and reduced membrane order, which in turn help keep FGFR2 in an inactive state [64]. Disruption of rafts using Chol- or SM-targeting agents selectively reduces non-activated VEGFR2 by promoting its lysosomal degradation, impairing ERK phosphorylation upon Vascular Endothelial Growth Factor (VEGF) stimulation. This highlights raft integrity as a determinant of cellular sensitivity to VEGF and of downstream signaling output. Although many RTKs localize within Chol- and sphingolipid-rich lipid rafts, others preferentially activate outside of microdomains. For instance, receptors such as EGFR and MET undergo ligand-induced dimerization and phosphorylation in non-raft regions, where higher membrane fluidity facilitates receptor mobility and clustering [68,69]. As a consequence of this, raft disruption can yield divergent outcomes. While some studies show that Chol depletion inhibits EGFR activation, others show enhanced ligand binding and EGFR hyperphosphorylation [68,70]. Rafts also regulate RTK activity by controlling ligand availability in the extracellular space. For example, Glypican-1, a heparan sulfate proteoglycan enriched in rafts, sequesters FGF2 and prevents its interaction with non-raft FGFRs [71]. In addition, Chol and GMs can exert chaperone-like effects, modulating receptor conformation and interfering with ligand recognition. Dietary lipids further influence raft composition. High-fat diets promote lipid oxidation and generate intermediates such as diglycerides (DGs) and ceramides. These lipids impair insulin signaling by activating kinases such as JNKs, IKKs, and PKC, which inhibit phosphorylated IRS-1, thereby blocking downstream activation [72]. Ceramide in particular induces insulin resistance by suppressing Akt activation and neutralizing IR signaling. During aging, loss of Chol from synaptic rafts leads to ligand-independent IR activation, contributing to brain insulin resistance [73]. Similarly, decreased Chol and GMs in neuronal rafts impair TRKB localization and ligand-dependent activation, thereby compromising neuronal survival, growth, and plasticity, hallmarks of neurodegenerative disease [74]. In Krabbe disease, accumulation of the cytotoxic lipid psychosine disrupts IGF1R signaling by preventing Akt phosphorylation. Psychosine alters membrane rigidity, curvature, and charge distribution, thereby blocking PI3K recruitment and reducing PIP3, mTORC2, and Akt levels [75]. Lipid raft alterations are also implicated in hepatic disease. Alcohol exposure modifies hepatocyte raft composition, increasing in membrane fluidity and impairing RTKs such as c-Met, with consequences for proliferation, morphogenesis, and survival [76]. Finally, raft localization can influence RTKs’ endocytosis and recycling. Raft-associated receptors are internalized via caveolae which can modify intracellular trafficking and receptor fate. Notably, rafts are frequently remodeled in cancer cells [77]. Tumors may contain up to 50% more Chol than normal tissues, expanding raft domains and hyperactivating RTK signaling, which fuel tumor progression. Modulating Chol levels can therefore rewire raft composition, RTK localization, and downstream signaling [78]. In epithelial cells, rafts dysregulation contributes to aberrant EGFR activation and resistance to tyrosine kinase inhibitors (TKIs) across cancer types [79]. Moreover, GM3, for example, regulates the localization of ErbB2 and EGFR within rafts without affecting phosphorylation, with major implications for epithelial cancers [80]. In glioblastoma, ELF4 overexpression alters phosphatidylcholine (PC), phosphatidylethanolamine (PE), and other PLs within rafts promoting RTK-dependent MAPK activation and tumorigenesis [77]. Lipid saturation also shapes membrane properties, increasing saturated and monounsaturated FAs, stiffens membranes and enhances lipid peroxidation, protecting cancer cells from oxidative stress and chemotherapy [81]. For example, oleic acid accumulation in membranes promotes EGFR activation through Src-mediated transactivation in breast cancer cells [82].

Among changes in membrane lipid balance, Chol depletion impacts receptor trafficking and endocytosis, for instance increasing surface expression of ErbB receptors in cancer cells. In EGFR, this enhances both ligand-dependent and ligand-independent activation [35]. Ret localization within rafts supports receptor activation and shields it from ubiquitination and degradation, as shown by increased Ret turnover upon raft disruption, a mechanism relevant to neurodegenerative diseases, cancer, or developmental disorders [83]. Moreover, deficiency of inositol polyphosphate 4-phosphatases B (INPP4B) increases PI(3,4)P2 levels in endocytic vesicles, altering RTKs recycling and promoting tumor progression. In a triple negative breast cancer, INPPB4 loss delays EGFR degradation and enhances recycling, sustaining oncogenic signaling [84]. Eventually, modulation of membrane lipid balance regulates the mechanical properties of the bilayer, inducing the membrane curvature which impacts the recruitment of receptors and other proteins. These intrinsically disordered proteins, described also as curvature-sensing proteins, may in turn further regulate the membrane curved structure [85]. Notably, the nontransmembrane receptor tyrosine kinase Fer, acting downstream to several RTKs and Src, owns an intrinsically disordered region that specifically binds to highly curved membranes, thus controlling where the effector is recruited [86]. Overall, the dynamic regulation of lipid rafts and membrane compartments is emerging as a critical factor in health and disease. Given its central role in controlling RTK signaling, endocytosis, and recycling, therapeutic strategies aimed at modulating raft composition and membrane rebalance are increasingly under investigation.

## 5. Lipid-Targeted Therapeutic Strategies

Lipid dysregulation plays a fundamental role in modulating RTKs’ activity across a broad spectrum of pathologies including cancer, neurodegenerative diseases, metabolic syndromes, and conditions associated with diet and aging. The integrity and composition of cellular membranes, particularly lipid rafts, critically influence RTK localization, clustering, and activation. Consequently, alteration in membrane lipids represents a major driver of aberrant RTK signaling in pathological conditions.

Several therapeutic approaches aim to modulate lipid metabolism and restore membrane lipid homeostasis in order to increase membrane fluidity, normalize the lipid raft assembly, and sensitize RTKs to pharmacological targeting. To this purpose, statins modulate membrane lipid composition and influence lipid rafts’ assembly through several ways. First, they block Chol biosynthesis by inhibiting the activity of 3-hydroxy-3-methylglutaryl coenzyme A reductase (HMG-CoA reductase) and impact other lipids metabolism, such as omega-3/-6 fatty acids and ceramide [87]. Second, statins directly interact with membrane lipid rafts. Based on their hydrophobicity degree, statins are able to penetrate the Chol-enriched phospholipid bilayer with variable efficacy, consequently destabilizing raft-associated proteins’ organization. For instance, more hydrophobic statins like cerivastatin penetrate deeper into the lipid raft bilayer, they disrupt lateral lipid packing, induce membrane thinning, and increase local curvature stress, consequently altering membrane mechano-properties. Accordingly, statins have been shown to increase membrane Young’s modulus, thickness and fluidity, thereby inducing mechanical heterogeneity within the bilayer [88,89]. Such perturbations can hinder the assembly of membrane-associated signalosomes, affecting RTKs signaling among others. Third, statin-induced changes in membrane composition also mislocalize lipidated proteins, which are covalently attached to lipid molecules for the correct membrane localization, consequently dampening their downstream signaling [87]. These therapeutic mechanisms suggest potential applications of statins across various pathological contexts with aberrant RTKs signaling like cancer, neurological disorders, and others. In cancer, for example, statins such as lovastatin and simvastatin disrupt EGFR- or IGF1R-containing lipid rafts, and enhance the efficacy of TKIs in prostate, breast, and lung cancer models [79]. In Huntington’s disease, statins’ disruption of lipid rafts affects TRKB clustering and AKT signaling [90]. Another promising class of agents is represented by synthetic anticancer alkylphospholipids (APLs), including edelfosine, miltefosine, and perifosine. These structurally related lipids accumulate in cellular membranes, interfere with lipid metabolism, and disrupt lipid-dependent RTK signaling. Their actions, particularly effective in metabolically active, proliferating cells, include inhibition of PC synthesis, suppression of MAPK/ERK, and PI3K/Akt pathways, and activation of the stress-activated protein kinase/JNK cascade, ultimately leading to apoptosis [91]. APLs’ integration into cell membranes, as observed with statins, alters protein packing and curvature elastic stress, thereby influencing elastic energy storage and the function of membrane-associated molecules [92]. This property is well characterized for amphiphilic molecules used as therapeutics, which physically integrate into the lipid bilayer tuning the membrane biophysics. Such capacity presents a valuable therapeutic strategy, enabling targeted modulation of membrane-bound receptors, such as RTKs, and signaling pathways implicated in cancer and degenerative diseases.

MβCD, a functionalized cyclodextrin, is used as a nanocarrier for therapeutic agents by forming complexes with drugs, where it enhances their solubility, stability, and bioavailability. Its distinctive cyclic structure enables the encapsulation of hydrophobic molecules, while its interaction with membrane Cholesterol can be exploited to modulate membrane properties and increase the permeability of specific drugs, and it is used in combination with TKIs for the effective treatment of several pathological conditions. Moreover, in vitro studies and clinical trials, MβCD has been extensively used for its capacity to deplete membrane Chol, consequently disrupting lipid rafts structures. For example, in Fibroblast COS-1 cell line, MβCD inhibits clathrin-mediated budding, reduces EGFR endocytosis, and prevents receptor phosphorylation [93]. Comparable effects have been reported for VEGFR2, where raft disruption promotes lysosomal degradation of the receptor and suppresses VEGF-A-mediated downstream signaling [94]. With a similar effect, agents like apolipoprotein A-I binding protein (AIBP), HDL, and their mimetics directly target Chol transporters such as ABCA1, increasing its efflux and destabilizing lipid rafts assembly, thus modulating RTK clustering and downstream signaling [95]. In addition to lipid-targeted strategies already implemented for cancer therapy, several developing approaches have not yet been associated with RTKs’ signaling regulation. For instance, inhibitors of the DHHC enzyme family, responsible for catalyzing protein palmitoylation, could modulate aberrant lipidation events. Their use, in combination with conventional anticancer treatments and RTKs inhibitors, may therefore enhance therapeutic efficacy by restoring proper receptor regulation. This was recently assessed for EGFR palmitoylation in mouse models, as assessed before, but still as to be translated in humans [96].

Beyond cancer, lipid-based interventions are increasingly explored in aging and metabolic disease. In the aging brain, where reduced Chol content in neuronal membranes enhances ligand-independent TRKB activation, liver X receptor (LXR) agonists promote Chol efflux via ABCA1, alleviating pathological signaling [97]. In insulin resistance and type 2 diabetes, accumulation of saturated FAs stiffens the plasma membrane, impairs IR activity, and disrupts downstream signaling. Strategies targeting ceramide biosynthesis or restoring membrane fluidity are under investigation to re-establish insulin sensitivity [98]. Moreover, diet-induced lipid imbalance (e.g., high-fat or high-Chol intake) can exacerbate RTKs’ activation in hepatocytes and endothelial cells, contributing to steatosis and atherosclerosis [99]. In this context, dietary interventions, through modulation of fat intake or supplementation with metabolites such as agmatine, an endogenous metabolic product of arginine with mitochondrial protective properties, have been proposed as strategies to slow down or even partially reverse these pathological conditions by restoring lipid homeostasis and improving fatty acid metabolism [99]. Finally, lipid-based nanocarriers are gaining attention as delivery RTK-targeted therapies and RNA-based drugs, enabling efficient transport across the blood–brain barrier or to metabolically active tissues, as in glioblastoma or nonalcoholic fatty liver disease. Overall, therapeutic strategies aimed at reprogramming lipid composition or disrupting lipid–RTK interactions hold significant promise, particularly in contexts where conventional TKIs show limited efficacy (Table 1).

## 6. Concluding Remarks

Despite significant progress in understanding how membrane lipids influence RTK behavior, our current knowledge remains limited. In particular, the contribution of RTK lipidation, among other post-translational modifications, is still poorly defined. Protein lipidation plays a crucial role in regulating protein trafficking and anchoring to specific membrane compartments. In this context, only a few examples of RTK lipidation have been described to date, showing that receptor function and activity can be modulated either directly or indirectly through their adaptor proteins. Although studies on other kinases and small proteins have provided valuable insights, these observations cannot be fully transferred to RTKs. Also, RTKs’ activation and trafficking are modulated by specific membrane lipid species such as Chol, SLs, and phosphoinositides, which interact with specific receptor sequences like the extracellular region, CARC/CRAC domains, and the JMD. Lipid binding modulates RTK activity in a context-dependent manner, modulating receptor dimerization and phosphorylation processes. However, the dynamic and condition-dependent nature of these interactions remains poorly characterized. Achieving high spatiotemporal resolution of lipid–RTK crosstalk, particularly in living cells and disease-relevant settings, continues to represent a major experimental challenge.

Furthermore, the assembly of lipid rafts, for instance, has been linked to both receptor activation and inhibition, as membrane compartmentalization can either facilitate or restrict RTK interactions. The contributions of lipid heterogeneity, local membrane curvature, and mechanical forces, factors that fundamentally shape receptor clustering and downstream signaling, are only beginning to be elucidated. Integrating lipidomics, structural biology, and advanced imaging approaches will be crucial to define how distinct lipid environments shape RTK function. Ultimately, a deeper understanding of this interplay could open new therapeutic strategies aimed at modulating membrane composition to selectively control pathological RTK signaling.

## Figures and Tables

**Table 1:** Lipid-targeted therapeutic approaches.

Therapeutic Strategy	Mechanism of Action	Effect	References
Statins	Inhibition of Cholesterol biosynthesis (HMG-CoA reductase), lipids metabolism, and lipid–lipid interaction. Penetration of membrane bilayer.	Lipid rafts’ instability leads RTKs signaling disruption and higher sensitivity to TKIs. Modification of membrane mechanical properties lead to alteration of protein function.	[79,87,90]
Methyl-beta-cyclodextrin	Forms inclusion complexes with Cholesterol, extracting it from membranes.	Used as nanocarrier for hydrophobic drugs, impairs lipid-dependent RTK signaling, and can trigger apoptosis	[93,100,101]
AIBP, HDL, and mimetics	Targeting of Chol transporters like ABCA1.	Increased Chol efflux, destabilization of lipid rafts, and reduced RTKs signaling	[95]
Alkylphospholipids	Insert into plasma membranes, disrupting lipid metabolism, phosphatidylcholine synthesis, and raft-associated signaling. Penetration of membrane bilayer.	Induction of cancer cell apoptosis and regulation of receptor trafficking, regulation of membrane-associated proteins.	[102,103]
LXR agonists	Activate LXRs to upregulate Cholesterol efflux transporters (ABCA1, ABCG1), reducing intracellular Cholesterol pools.	Reduce lipid accumulation and membrane rigidity; potential to modulate RTK localization.	[97,104,105]
Ceramide inhibitors	Modulation of ceramide biosynthesis and membrane composition.	Restoration of insulin sensitivity.	[98,106,107]
DHHC enzymes inhibitors	Modulation of proteins’ lipidation events.	Higher sensitivity to anticancer therapies, increasing sensitivity to TKIs.	[36,96]
Diet modulation and metabolites intake	Regulation of fatty acids metabolism and mitochondrial stability.	Restore lipid homeostasis and membrane composition, indirectly influencing RTK activation and downstream signaling.	[99]
Lipid-based nanocarriers	Cross blood–brain barrier and metabolic active tissues.	Delivering of RTK-targeted drugs or RNA-based therapies	[108,109,110]

## Data Availability

No new data were created or analyzed in this study.

## Abbreviations

Apolipoprotein A-I Binding Protein (AIBP), Alkylphospholipids (APLs), Conserved amino acid consensus sequences (CARC), Cholesterol Recognition Amino Acid Consensus (CRAC), Cholesterol (Chol), Diglycerides (DGs), Epidermal Growth Factor (EGF), Epidermal Growth Factor Receptor (EGFR), Fatty Acids (FAs), Fibroblast Growth Factor (FGF), Fibroblast Growth Factor Receptor (FGFR), Fibroblast Growth Factor Receptor Substrate 2α (FRS2α), Gangliosides (GMs (1,2,3)), G Protein-coupled Receptors (GPCRs), Glycerophospholipids (GPLs), Glycosphingolipids (GSLs), Juxtamembrane domain (JMD), Insulin-like Growth Factor 1 Receptor (IGF1R), Inositol polyphosphate 4-phosphatasis B (INPP4B), Inositol triphosphate (IP_3_), Kinase Domain (KD), Lipid-derived electrophiles (LDE), Lysophosphatidic Acid (LPA), Lipid Transfer Proteins (LTPs), Liver X receptor (LXR), Methyl-β-cyclodextrin (MβCD), Mono- Di- Triacylglycerol (M- D- TAGs), OSBP-Related Protein (ORPs), Oxysterol-binding Protein (OSBP), Phosphatidylcholine (PC), Phosphoinositide-dependent kinase 1 (PDK1), Phosphatidylethanolamine (PE), Pleckstrin Homology (PH), Pleckstrin Homology domain Leucine-rich repeat Protein Phosphatase (PHLPP), Phosphoinositide 3-kinase (PI3K), Phosphatidylinositol-4,5-bisphosphate (PIP2), Phosphatidylinositol-3,4,5-triphosphate (PIP3), Phosphatidylinositol-Phosphates (PIPs), Phospholipase C gamma (PLCγ), Phospholipids (PLs), Phox Homology (PX), Phosphatidylserine (PS), Polyunsaturated fatty acids (PUFA), Tyrosine Kinase Receptors (RTK), Sphingosine-1-Phosphate (S1P), Sphingolipids (SLs), Sphingomyelin (SM), Tyrosine Kinase Inhibitors (TKIs), Transmembrane domain (TMD), Tropomyosin-related kinase B (TRKB), Vascular Endothelial Growth Factor (VEGF), Vascular Endothelial Growth Factor Receptor (VEGFR).
